# Insight into the Molecular Mechanism of Surface Interactions of Phosphatidylcholines—Langmuir Monolayer Study Complemented with Molecular Dynamics Simulations

**DOI:** 10.1021/acs.jpcb.3c06810

**Published:** 2024-02-06

**Authors:** Anna Chachaj-Brekiesz, Jan Kobierski, Anita Wnętrzak, Patrycja Dynarowicz-Latka, Patrycja Pietruszewska

**Affiliations:** † Faculty of Chemistry, Jagiellonian University, Gronostajowa 2, 30–387 Kraków, Poland; ‡ Department of Pharmaceutical Biophysics, Faculty of Pharmacy, Jagiellonian University Medical College, Medyczna 9, 30–688 Kraków, Poland

## Abstract

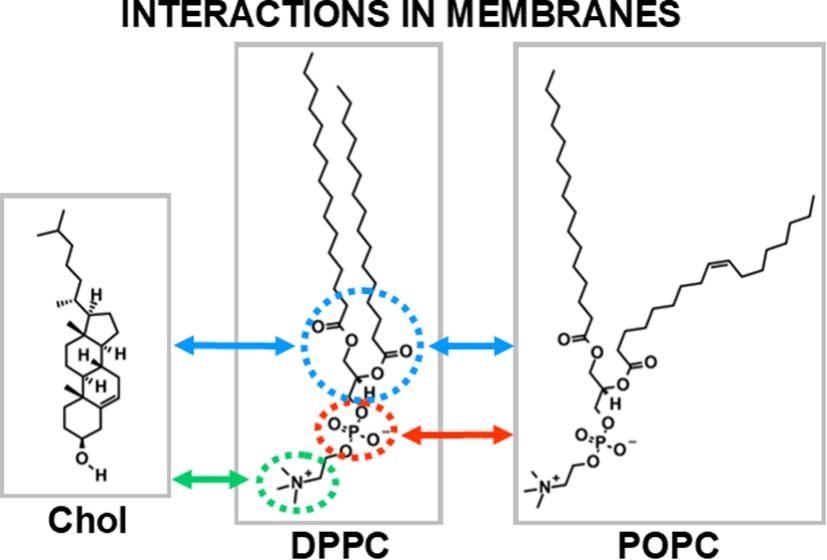

Mutual interactions between components of biological membranes are pivotal for maintaining their proper biophysical properties, such as stability, fluidity, or permeability. The main building blocks of biomembranes are lipids, among which the most important are phospholipids (mainly phosphatidylcholines (PCs)) and sterols (mainly cholesterol). Although there is a plethora of reports on interactions between PCs, as well as between PCs and cholesterol, their molecular mechanism has not yet been fully explained. Therefore, to resolve this issue, we carried out systematic investigations based on the classical Langmuir monolayer technique complemented with molecular dynamics simulations. The studies involved systems containing 1,2–dipalmitoyl–*sn*–glycero–3–phosphocholine (DPPC) analogues possessing in the structure one or two polar functional groups similar to those of DPPC. The interactions and rheological properties of binary mixtures of DPPC analogues with 1–palmitoyl–2–oleoyl–*sn*–glycero–3–phosphocholine (POPC) and cholesterol were compared with reference systems (DPPC/POPC and DPPC/cholesterol). This pointed to the importance of the ternary amine group in PC/cholesterol interactions, while in PC mixtures, the phosphate group played a key role. In both cases, the esterified glycerol group had an effect on the magnitude of interactions. The obtained results are crucial for establishing structure–property relationships as well as for designing substitutes for natural lipids.

## 1 Introduction

In biological systems, interactions between membrane components and with external molecules determine the membrane’s biophysical properties and the physiological effects of bioactive compounds. The method used to study such interactions is the Langmuir monolayer technique,^1^ the successful applications of which in this area have been thoroughly discussed.^2^ It enabled, among others, to find molecular targets of many bioactive molecules (e.g., drugs, for example, antitumor lipids, abbr. antitumor lipids (ATLs),^3^ antimicrobial agents,^4^ or food supplements–unsaturated fatty acids or stanols^5^), their mechanism of action^6−9^ and the reason for their selectivity^10,11^ or toxicity.^12−15^ It also allowed us to understand how changing the composition of the membrane and the mutual proportions of its components (e.g., cholesterol-to-phospholipid ratio; sphingolipid content; unsaturation of the phospholipids fatty acids) changes the stiffness of the membranes. Such variations occur in the physiological conditions (for example, when following a specific diet,^16^ taking various medications,^17^ or even during physical activity^18^), as well as are characteristic for the development of many pathological effects (related to cancer^19^ or other diseases, like anemia,^20^ or in poisoning (e.g., with ethanol^21^)).

Studies of interactions between membrane components or between the membrane and the biomolecule are based on the calculation of particular qualitative and quantitative parameters^1,22^ derived from the experimental surface pressure–molecular area (π–*A*) isotherms. The most informative are values of the thermodynamic excess functions of mixing, among which the most frequently determined is excess Gibbs free energy of mixing (Δ*G*^exc^), as it allows one to determine the type (attractive/repulsive) and strength of interactions and the thermodynamic stability of the investigated system.

Additionally, molecular dynamics (MD) simulations can be used to elucidate interactions between lipids in monolayers, offering a unique perspective into the systems of these molecules. By employing implementations of theoretical models, it is possible to study components of interactions between different types of molecules, even at the atomic level. These simulations provide valuable information about the structural arrangement, conformation, and energy distribution of the investigated lipid layers. A precise understanding of these aspects is essential to unraveling the fundamental principles underlying biological processes and membrane–related phenomena.

Studies of interactions between membrane components usually involve mixtures of lipids, for example, various phospholipids and sterols. In the case of membrane–biomolecule interactions, the molecule under study can also have a lipid-like structure (e.g., ATLs or oxysterols). Although there is extensive literature in this field, several key issues remain unclear. Namely, it was demonstrated that polar interactions contribute more significantly to the overall molecular interactions compared to hydrophobic interactions;^23−27^ however, the molecular origin of these interactions is not fully understood. This applies especially to phospholipids, which have several polar groups in their structure, e.g., 1,2-dipalmitoyl-*sn*-glycero-3-phosphocholine (DPPC). Knowing the type of polar group with the greatest contribution to the interactions would allow for the possible use of substitutes with increased stability in laboratory research.

**Figure 1:**
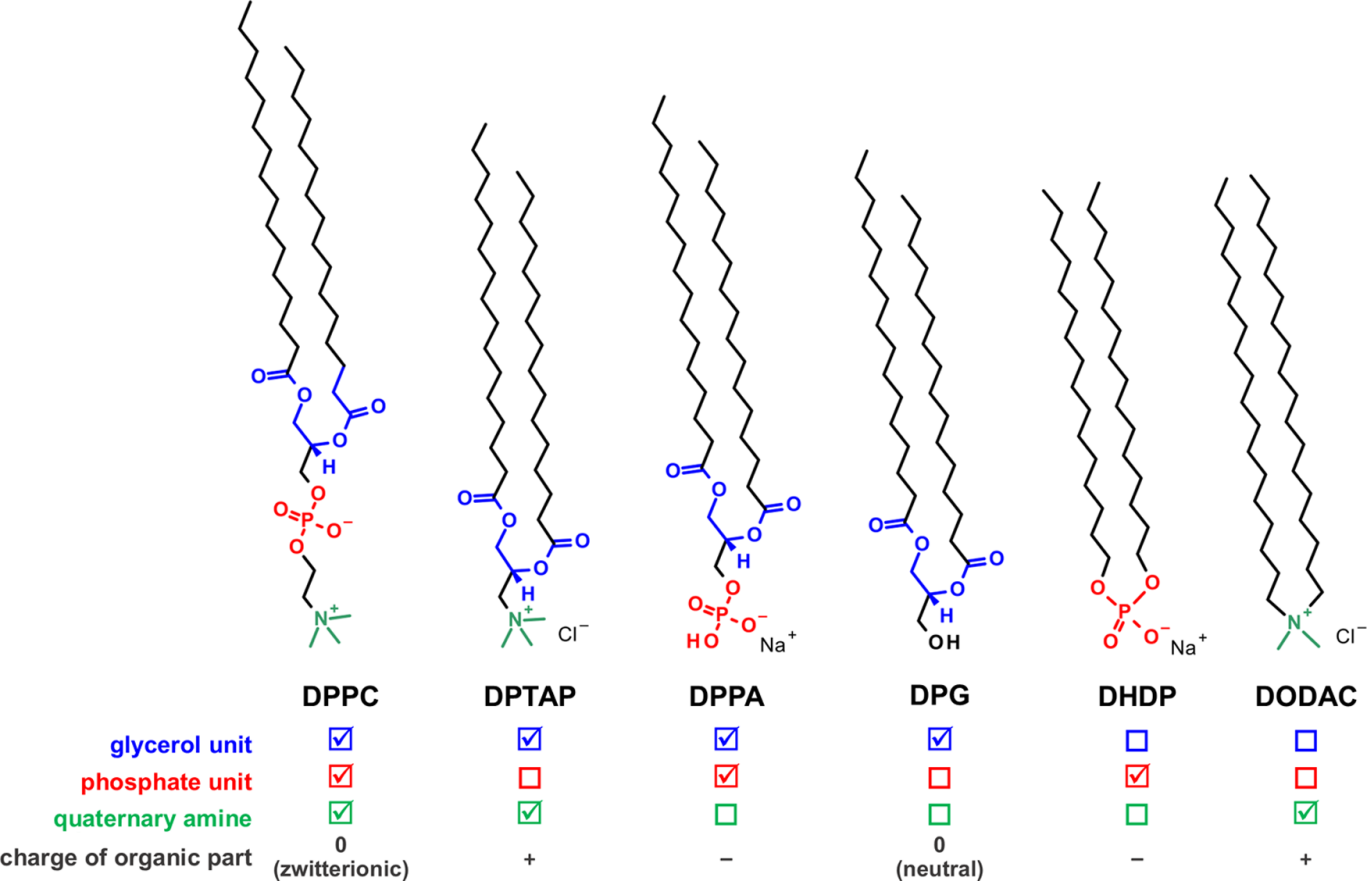
Chemical structures of the investigated compounds along with a comparison of the functional groups present in their molecules (symbol means the presence, while □ means the absence of the selected functional group). Abbreviations: DPPC—1,2–dipalmitoyl–*sn*–glycerol–3–phosphocholine; DPTAP—1,2–dipalmitoyl–3–trimethylammonium–propane chloride; DPPA—sodium 1,2–dipalmitoyl–*sn*–glycero–3–phosphate; DPG—1,2–dipalmitoyl–*sn*–glycerol; DHDP—sodium dihexadecylphosphate; DODAC—dimethyldioctadecylammonium chloride.

The aim of the work was to obtain information about the molecular origin of the interactions that are key to the stability and rheological properties of the membrane. For this purpose, we carried out systematic research based on classical thermodynamic analysis of π–*A* isotherms and MD simulations performed for a surface pressure of 35 mN/m, as it reflects the conditions in biomembranes.^28,29^ For our experiments, we chose lipids responsible for maintaining the physicochemical properties of the biomembrane: cholesterol (Chol) and phosphatidylcholines (PC).^30^ As phosphatidylcholines with different chain lengths and unsaturation account for about 50% (in mass) in eukaryotic cells,^30^ we assumed that crucial for the membrane biophysical properties are interactions between Chol and PC as well as between different PCs. Taking this into account, we have chosen the representative of unsaturated (1–palmitoyl–2–oleoyl–*sn*–glycero–3–phosphocholine (POPC)) and saturated (1,2–dipalmitoyl–*sn*–glycero–3–phosphocholine (DPPC)) phosphatidylcholines. DPPC analogues (compounds with saturated chains (mainly hexadecyl) in the hydrophobic part and having one or two functional groups analogous to those of DPPC in the hydrophilic head) are commercially available. In this way, a full library of DPPC analogues having at least one of the DPPC functional groups as their polar heads is readily available (Figure 1). Moreover, by using an appropriate subphase, the same ionization of hydrophilic groups as that occurring in DPPC was achieved. The library of compounds mentioned above was investigated in binary mixtures with Chol and POPC. In the final step of our research, we employ MD simulations to delve into the complex processes occurring within lipid monolayers, shedding light on the nuanced mechanisms directing their dynamics. This has allowed us to explain the results of our experiments.

## 2 Materials and Methods

### 2.1 Materials

Dimethyldioctadecylammonium chloride (DODAC) was obtained by ion exchange from dimethyldioctadecylammonium bromide (purity >99%, Avanti Polar Lipids) spread on aqueous subphase containing NaCl (0.1 mol/L). The choice of this particular quaternary alkylammonium salt was dictated by the fact that among the dimethyl dioctadecyl ammonium salts, the one containing the chloride anion has the characteristic of the π–*A* isotherm most similar to DPPC, showing the LE–LC phase transition in a similar range of surface pressures.^31^ DHDP (sodium dihexadecylphosphate) was obtained from dihexadecylphosphate (purity >99%, Avanti Polar Lipids) spread on an aqueous subphase containing NaOH (0.001 mol/L). Other compounds: 1,2–dipalmitoyl–*sn*–glycero–3–phosphocholine (DPPC), 1–palmitoyl–2–oleoyl–*sn*–glycero–3–phosphocholine (POPC), cholesterol (Chol), 1,2–dipalmitoyl–*sn*–glycerol (DPG), sodium 1,2–dipalmitoyl–*sn*–glycero–3–phosphate (DPPA), 1,2–dipalmitoyl–3–trimethylammonium–propane chloride (DPTAP) of high purity (>99%) were supplied by Avanti Polar Lipids and used as received. Chloroform stabilized with ethanol (HPLC purity, Sigma–Aldrich) was used to prepare the spreading solutions. Unless otherwise indicated, ultrapure water from an HLP demineralizer (Hydrolab) with a conductivity of <0.06 μS/cm was used as the subphase.

### 2.2 Methods

#### 2.2.1 Langmuir Monolayer Technique

Individual chemicals were dissolved in chloroform to obtain stock solutions with a concentration of 0.2–0.3 mg/mL. To prepare binary monolayers, the appropriate volume of stock solutions was measured by using a microsyringe (Hamilton, maximum capacity 250 ± 2.5 μL) and mixed in a vial. Then, the selected (one- or two-component) chloroform solution was carefully deposited dropwise at the appropriate subphase (water, aqueous NaCl, or NaOH) placed in a double-barrier Langmuir trough (KSV 2000, total area of 700 cm^2^). The film was equilibrated for 5 min, and surface pressure measurements–molecular area (π–*A*) isotherms were started at a compression speed of 20 cm^2^/min. The subphase temperature was maintained at 20 ± 0.1 °C using a cooling unit (Julabo) with a circulating water system. Surface pressure measurement was performed using the Wilhelmy method using a strip of ashless filter paper (Whatman Chr1). On the basis of experimental π–*A* isotherms for binary monolayers, the values of excess Gibbs free energy of mixing (Δ*G*^exc^) were calculated, reflecting the strength of mutual interactions and the affinity of its components to each other, using equation^22^
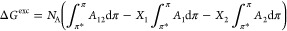
1where *N*_A_ is Avogadro number, *A*_1_, *A*_2_, and *A*_12_ are area per molecule values read from isotherms of pure substances (1 and 2) and their mixtures, and *X*_1_ and *X*_2_ are the mole fractions of substances 1 and 2 in the mixture. Δ*G*^exc^ provides information on whether the particular interaction is energetically favorable (negative values) or not (positive values), while for Δ*G*^exc^ = 0, ideal mixing (or immiscibility) occurs.^22^

Additionally, in order to characterize the mechanical properties of monolayers, surface compressional modulus (*C*_s_^–1^) values were calculated according to the equation
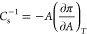
2*C*_s_^–1^ values below 25 mN/m suggest that the film is in a low–density liquid phase; the ranges of 25–50 mN/m and 100–250 mN/m are characteristic for the liquid expanded and liquid condensed states, respectively, while for *C*_s_^–1^ above 500 mN/m, the film is in the solid state.^1^

#### 2.2.2 Theoretical Calculations

Molecular dynamics simulations were performed using the Amber22 package.^32^ Three simulated systems consisted of two rectangular symmetric monolayers, each monolayer having 128 lipid molecules (64 POPC molecules and 64 DPPC, DPPA, or DHDP molecules), and 15,000 water molecules were built in Packmol software.^33^ Sodium ions were added to neutralize the POPC/DPPA and POPC/DHDP systems. Periodic boundary conditions were utilized. We used the lipid21^34^ force field for lipids and the 3–charge, 4–point OPC model for water.^35^ For DHDP, electrostatic potentials were calculated quantum mechanically using the Gaussian 16.^36^ Atomic point charges were calculated in the Antechamber program^37^ using the RESP model.^38^ The missing parameters were taken from the GAFF2 force field.^39^ The energy of the systems was minimized by 10,000 steps. The systems were then equilibrated by 75,000 steps with a 0.001 ps time step, followed by 150,000 steps with a 0.002 ps time step. Production calculations were carried out in an isothermal–isobaric ensemble with constant surface tension corresponding to a surface pressure of 35 mN/m (NPγT) with a 0.002 ps time step. The temperature was set at 20 °C, and a Langevin thermostat was used. Berendsen aerostat was used to control the pressure at 1 bar. The simulation was carried out for 600 ns of the system evolution. The last 100 ns were used for analysis. The results were analyzed in the CPPTRAJ program.^40^

## 3 Results and Discussion

### 3.1 Surface Behavior of DPPC and its Analogues

**Figure 2:**
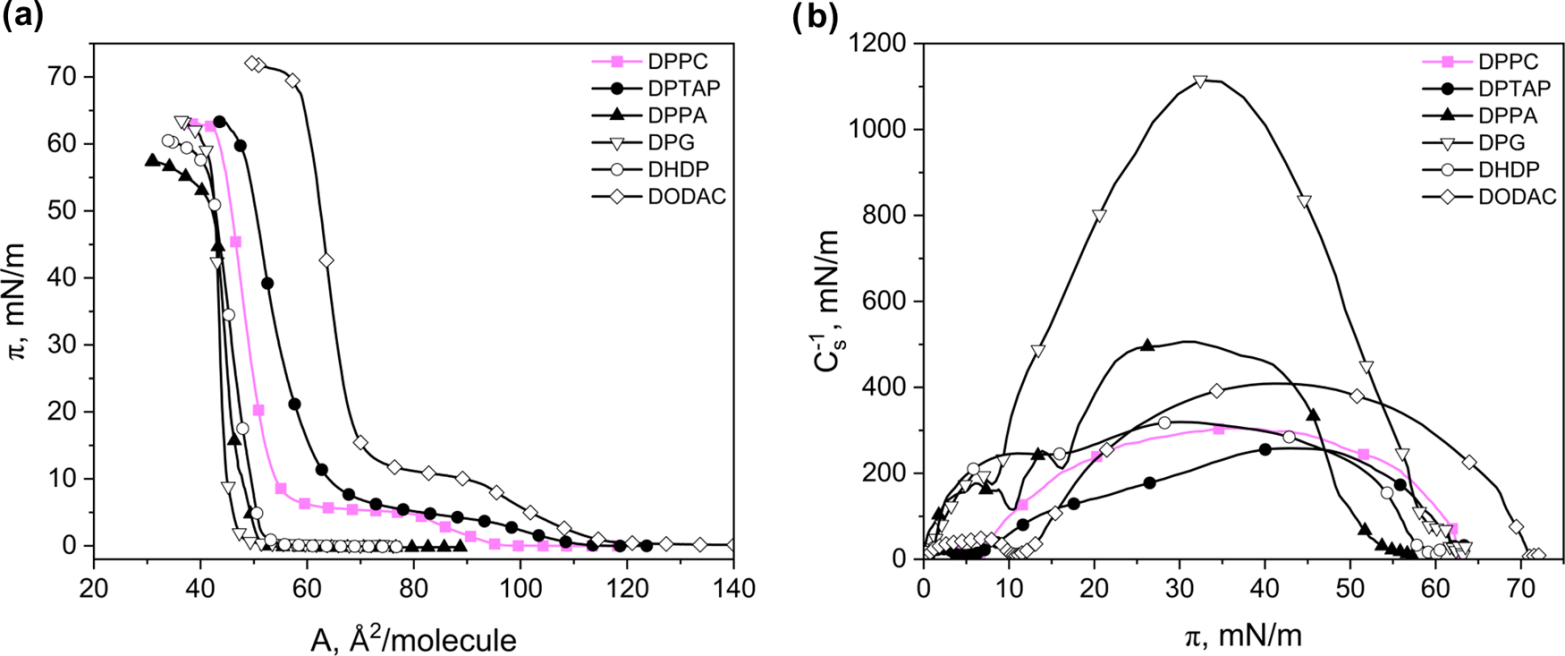
Surface behavior of DPPC and its analogues in Langmuir monolayers: (a) experimental surface pressure–area per molecule isotherms measured at 20 °C and (b) calculated compressibility moduli–surface pressure curves.

DPPC is one of the most studied phospholipids, and the origin of its membrane activity was attributed to its unique surface and phase behavior. Namely, this lipid has the ability to arrange itself at the phase boundary into two types of stable two-dimensional (2D) states with different molecular packing and conformation. In experimental π–*A* isotherms most pronounced is a wide plateau region at around 5 mN/m reflecting the coexistence region of low-pressure liquid expanded (LE) state and high-pressure liquid condensed (LC) phase.^41^ The first question, which turned out to be crucial for our research, was whether modification of the hydrophilic region of the molecule would affect the phase transition, which is mainly related to changes in the order of apolar hydrocarbon chains.^41^ Therefore, we measured π–*A* curves for DPPC analogues and compared them with the results for DPPC (Figure 2a). To gain insight into the mechanical properties, the surface compression modulus was also calculated and plotted against the surface pressure (Figure 2b).

The measured curves were in accordance with the literature isotherms of DPTAP,^42^ DPPA,^43^ DPG,^44^ DHDP^45^, and DODAC.^46^ Interestingly, molecules without a ternary amine group in their structure (DPG, DPPA, and DHDP) are characterized by experimental π–*A* dependencies of similar lift-off point values (49, 51, and 53 Å^2^/molecule, respectively), although they differ from the one observed for DPPC. In the course of the compression, no inflections are visible until the collapse, occurring above 50 mN/m (59, 52, and 57 mN/m, respectively). The isotherms of these compounds differ in slope, which is also reflected in compressional moduli values. In particular, for a surface pressure of 35 mN/m, these films have *C*_s_^–1^ values of approximately 1107 (for DPG), 490 (for DPPA), and 313 mN/m (for DHDP). On the contrary, compounds possessing in their structure the ternary amine group (DPTAP and DODAC) have experimental curves with strikingly greater lift-off point values (at 111 and 117 Å^2^/molecule, respectively), which are close to that for DPPC (98 Å^2^/molecule) and show noticeable plateau regions. The plateau in both isotherms is not as well-defined as for DPPC (it is not perfectly horizontal); however, the *C*_s_^–1^ values for pressures at the plateau reach a minimum close to 0 mN/m, similar to DPPC. The latter, together with (i) maximum values of *C*_s_^–1^ for phase below transition falling in the range characteristic for LE phase (26 and 48 mN/m for DPTAP and DODAC, respectively) and (ii) *C*_s_^–1^ values for the phase above the plateau corresponding to the LC phase; confirms that we deal with the LE–LC phase transition. This was also supported by Brewster angle microscopy observations.^42,47^ Therefore, based on the analysis of the π–*A* and *C*_s_^–1^–π curves, it can be concluded that the ternary amine group promotes the induction of surface pressure–dependent conformational changes in molecules with hexadecyl hydrocarbon chains.

### 3.2 Molecular Origin of DPPC Interactions with Cholesterol

**Figure 3:**
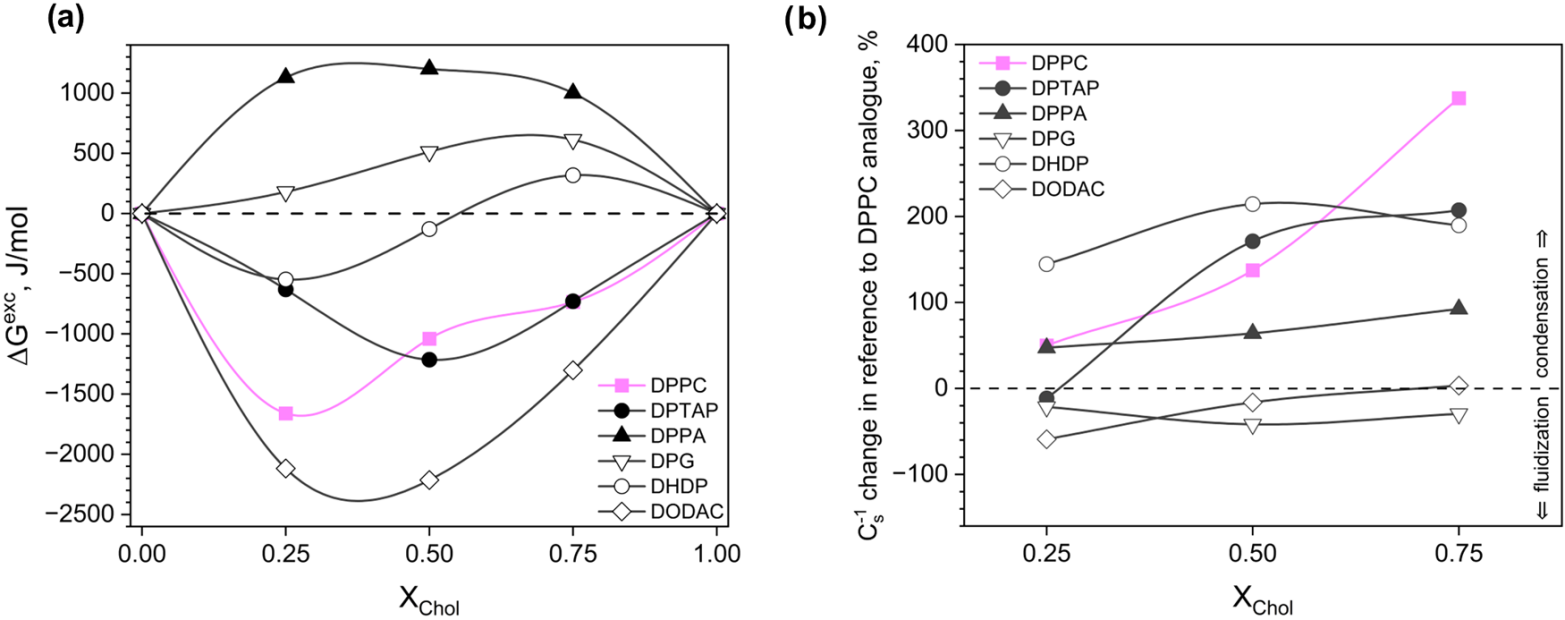
DPPC and its analogues in binary films with Chol: (a) the excess Gibbs free energy of mixing and (b) changes in compressibility moduli values with respect to a monolayer of the respective DPPC analogue versus film composition at a surface pressure of 35 mN/m.

In the next step of our research, we were interested in how the surface activity and structure of DPPC analogues are reflected in interactions with one of the main membrane lipids — cholesterol. For this purpose, we investigated the DPPC analogues in binary mixtures with cholesterol (π–*A* and *C*_*s*_^–1^–π curves for these systems are presented in Figures S1 and S2 in the Supporting Information). Based on π–*A* isotherms, Δ*G*^exc^ values were calculated and plotted for the chosen surface pressure values (Figure S3 in the Supporting Information). Next, the Δ*G*^exc^–*X*_Chol_ curves obtained for each DPPC analogue at 35 mN/m were compared in Figure 3a.

As can be seen, for the DPPC/Chol mixtures, deviations from the ideal behavior suggesting dose-dependent attractive interactions can be found. Their strongest magnitude is approximately equal to −1660 J/mol (for *X*_Chol_ = 0.25) and is associated with the formation of DPPC/Chol surface complexes of a 3:1 stoichiometry.^48^ The results for binary mixtures indicate diverse behavior of DPPC analogues in binary films with cholesterol, starting from strong repulsion (DPPA) through weak or ideal interactions (DPG and DHDP) to attraction (DPTAP and DODAC). From a structural point of view, it appears that the ternary amino group, which is a moiety present in the structures of both DPTAP and DODAC, is crucial for the occurrence of attractive interactions with cholesterol. For these compounds, the minima in the Δ*G*^exc^–*X*_Chol_ curves occur at approximately *–*1215 J/mol (for *X*_Chol_ = 0.50 in the DPTAP/Chol film) and −2214 J/mol (for *X*_Chol_ = 0.50 in the DODAC/Chol film), suggesting strong affinity between molecules. Indeed, theoretical studies have shown that almost every ternary amino group of PC forms charge pairs with the hydroxyl group of Chol.^49^ The above interaction is considered to be the most probable in the Chol/DPPC systems, while the second important one is the H*-*bond between the hydroxyl group of Chol and the carbonyl groups of PC.^50^ Based on our results, it can be noticed that the existence or lack of glycerol backbone and ester groups in the structure of surface active compounds modifies the magnitude of interactions with cholesterol. Therefore, the secondary role of the carbonyl group is experimentally confirmed herein. At the same time, Chol does not participate preferentially in interactions with the phosphate group, as suggested by similar Δ*G*^*exc*^ values for the Chol/DPPC and Chol/DPTAP systems. This issue has also been demonstrated in several MD simulations^51−53^ and energy minimalization studies.^54^

Since cholesterol is crucial to maintain the proper ordering and condensation of the biomembrane, we decided to analyze its effect on monolayers of DPPC and its analogues. Figure 3b shows the percent change in the compressibility moduli values of the binary films in relation to *C*_s_^–1^ of the monolayers from the corresponding DPPC analogues. It is well-known that cholesterol exerts a condensing effect on phosphatidylcholine films, and what is important, the degree of condensation is strongly related to the increase in cholesterol concentration.^48,55^ In our case, the condensation of the DPPC film for *X*_Chol_ = 0.25 is equal to 50%, while for *X*_Chol_ = 0.75, it increases to 337%. As can be seen, the effect of cholesterol on films from the DPPC analogues investigated herein is varied. For example, DODAC and DPG monolayers are slightly fluidized (up to 60%) upon the addition of cholesterol. In contrast, the stiffness of films from compounds containing phosphate groups (DHDP and DPPA) is increased by cholesterol in the entire range of the investigated mole fractions, but the effect is not as dose-dependent as in the case of the DPPC/Chol system. In turn, a small decrease (11%) in *C*_s_^–1^ is observed for the DPTAP film with the lowest cholesterol content, while for higher doses, significant condensation occurs (up to 207%).

The condensing effect of cholesterol on films from saturated phosphatidylcholines, exemplified by DPPC, has been widely studied using theoretical MD approaches (for a review, see ref (50)). It has been shown that this effect results from (i) an increase in the conformational order of the hydrocarbon chains (reduction of the number of gauche defects) and (ii) a decrease in the surface area occupied by PC molecules in mixed monolayers compared to pure PC monolayers.^50^ Such phenomenon was found to be related to the fact that the PC hydrocarbon chain packing is closer when they are at the same depth in the surface layer as the cholesterol ring system.^50,56^ The results from our experiments suggest that the degree of condensation of monolayers from DPPC analogues with the same hydrocarbon chain length is related to the type of polar groups in their structure. Namely, cholesterol-induced condensation occurs in the entire range of concentrations for films containing DHDP (having a phosphate group) and DPPA (with a phosphate and glycerol moiety). Therefore, it can be assumed that the phosphate group is crucial for the occurrence of cholesterol-induced condensation of DPPC films. As indicated by the interaction analysis performed here (based on Δ*G*^exc^) and MD simulations,^50^ the phosphate group of DPPC is involved in polar interactions with water rather than with cholesterol molecules. This, in turn, may suggest that DPPC analogues containing the phosphate group are anchored in water approximately at the same depth as DPPC, and therefore, their hydrocarbon chains may be packed more tightly.

### 3.3 Molecular Origin of DPPC Interactions with Other Phosphatidylcholines on the Example of POPC

**Figure 4:**
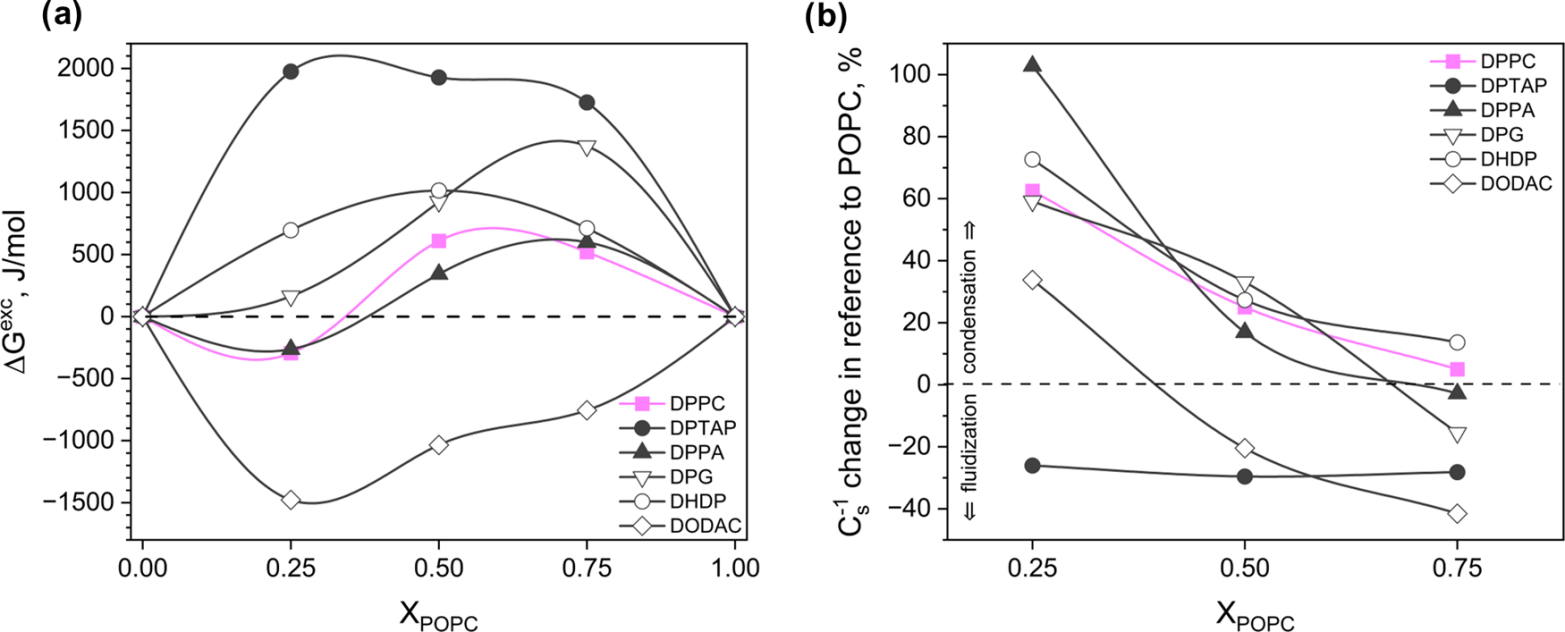
DPPC and its analogues in binary films with POPC: (a) excess Gibbs free energy of mixing and (b) changes in compressibility moduli values in reference to the POPC monolayer versus film composition at a surface pressure of 35 mN/m.

Our further experiments aimed to obtain information on how the surface activity and structure of DPPC analogues influence interactions with the main lipid of the fluid membrane matrix — POPC. For this purpose, we investigated DPPC analogues in binary mixtures with POPC (π–*A*, *C*_s_^–1^–π, and Δ*G*^exc^–*X*_POPC_ curves for these systems are presented in Figures S4–S6 in the Supporting Information). Based on these data, the Δ*G*^exc^*–X*_POPC_ curves and the *C*_s_^–1^ change in reference to POPC were compared in Figure 4 for a surface pressure of 35 mN/m.

The Δ*G*^exc^ values for the DPPC/POPC mixture suggest that in this system, the interactions of differential character occur (weak attractive for *X*_POPC_ = 0.25 and weak repulsive for a greater POPC content).^57^ Therefore, it can be perceived that DPPC does not show a preferential affinity for POPC. However, mixtures of POPC with DPPC analogues having a quaternary amino group are characterized by very strong interactions of an attractive (for DODAC) or repulsive (for DPTAP) nature in the entire range of mole fractions. Slightly weaker (but repulsive) interactions were found in systems containing DPG and DHDP. Finally, among all binary mixtures tested, an approximately similar course of the Δ*G*^exc^–*X*_POPC_ relationship as for DPPC/POPC can be observed for DPPA/POPC. However, the magnitude of interactions in the equimolar mixture is, in this case, weaker (Δ*G*^exc^ is approximately 267 J/mol lower). This suggests that the cooperation of the phosphate group with the glycerol skeleton plays a key role in the mutual interactions between phosphatidylcholines. The importance of the latter is evidenced by the fact that the interactions of DHDP molecules possessing only a phosphate group in the structure are slightly stronger and repulsive.

Moving on to the analysis of mechanical properties, it can be seen that the addition of DPPC to the POPC film causes gradual, dose-dependent condensation and stiffening. This effect was attributed to the increase in conformational order and lipid chain packing caused by an increase in the concentration of the zig–zag conformation characteristic of saturated (DPPC) chains.^58^ What is interesting is that although all of the investigated DPPC analogues possess similar saturated hydrocarbon chains in their structure, they affect the rheology of films mixed with POPC in a different way. Namely, monolayers with compounds containing the ternary amine group (DPTAP and DODAC) are significantly more fluidized compared to the DPPC/POPC system. Meanwhile, DPG/POPC, DHDP/POPC, and DPPA/POPC systems show approximately the same rheological behavior as the DPPC/POPC mixture, with two exceptions: (i) a small dose of DPG exerts a fluidizing effect of about 15%, while (ii) DPPA in concentrations above 0.75 seems to exert about 30% greater condensation compared to DPPC/POPC.

**Figure 5:**
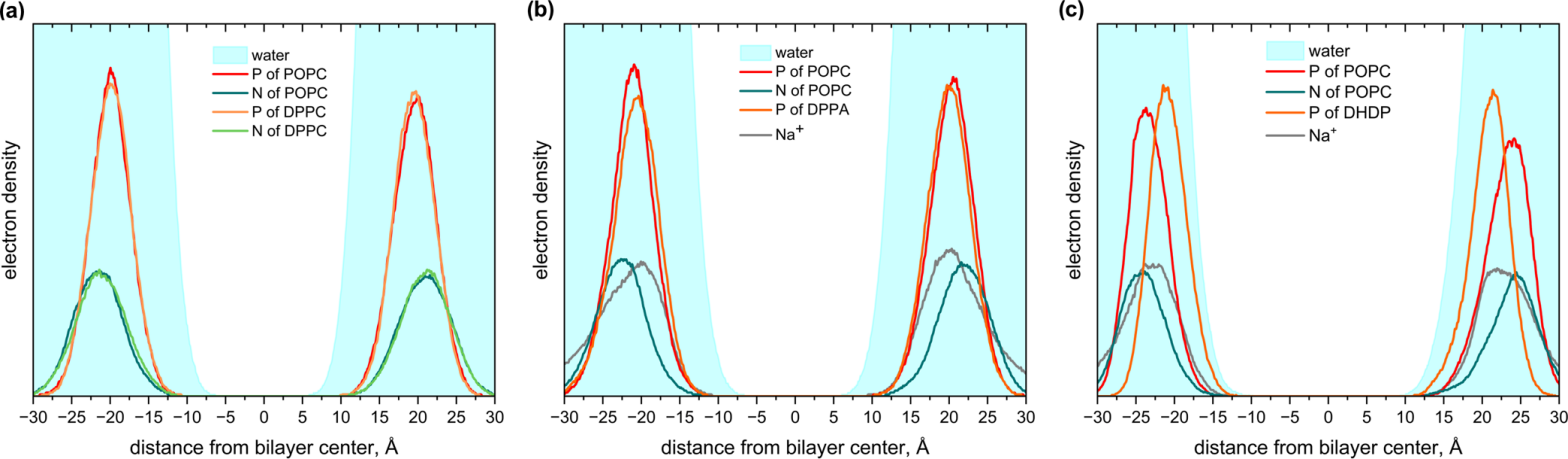
Electron density profiles across systems simulated with MD: (a) DPPC/POPC, (b) DPPA/POPC, and (c) DHDP/POPC.

The experimental results mentioned above suggest that the phosphate and glycerol moiety participate collectively in interactions between DPPC and POPC, whereas the amine group is noninteracting in this system. To gain better insight into this issue, we performed molecular dynamics simulations for the following systems mixed in a 1:1 ratio: reference DPPC/POPC (this system had been previously investigated using molecular dynamics simulations^58^), DPPA/POPC (as DPPA is a DPPC analogue that possesses a phosphate and glycerol moiety), and DHDP/POPC (as DHDP is a DPPC analogue that possesses a phosphate moiety). Figure 5 shows electron density profiles for simulated systems, which point to a strong similarity between the DPPC/POPC and DPPA/POPC systems. The phosphorus atoms of the phosphate groups in POPC, DPPC, and also DPPA are immersed in water to a depth of about 9 Å. At the same time, the electron density profile for the DHDP/POPC system is different. Here, the lack of an ester moiety in the DHDP molecule prevents it from anchoring so deep in water, resulting in the phosphorus atom of the phosphate group of DHDP being located at a depth of approximately 4 Å.

**Figure 6:**
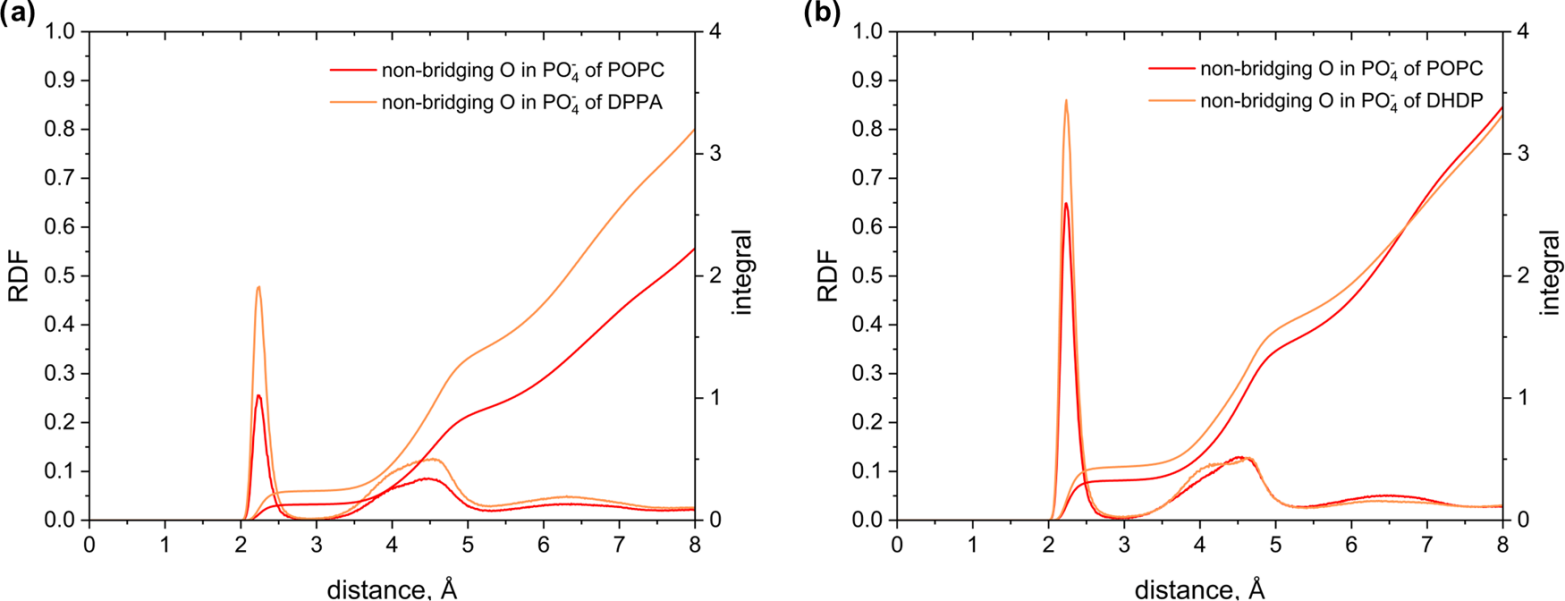
Radial distributions function of Na^+^ ions around the nonbridging oxygens in a phosphate group of the (a) DPPA/POPC and (b) DHDP/POPC systems.

A wider distribution of phosphate groups in the DHDP/POPC system affects the distribution of sodium counterions, which may result in a change in electrostatic interactions. To investigate the coordination of counterions with functional groups, we determined the radial distribution function (RDF) of the pairs sodium cation–nonbridging oxygen in the phosphate group in DPPA/POPC and DHDP/POPC systems (Figure 6). The RDF plots show that the cations are distributed around the phosphate groups with two peaks, one narrow and one broad, at a distance of approximately 2.2 and 4.5 Å from the oxygen, respectively, which is consistent with values previously determined in the molecular dynamics of DPPC systems^59^ and corresponds to the coordination shells. In the DPPA/POPC system, more cations accumulate around the DPPA phosphate group than around the POPC phosphate group. In the case of the DHDP/POPC system, in turn, similar integrals of the RDF functions for POPC and DHDP suggest a similar distribution of cations around these lipids. Although the integral of RDF for DHDP does not differ significantly from that for DPPA, the integrals of the RDF for POPC differ dramatically for these two systems.

**Figure 7:**
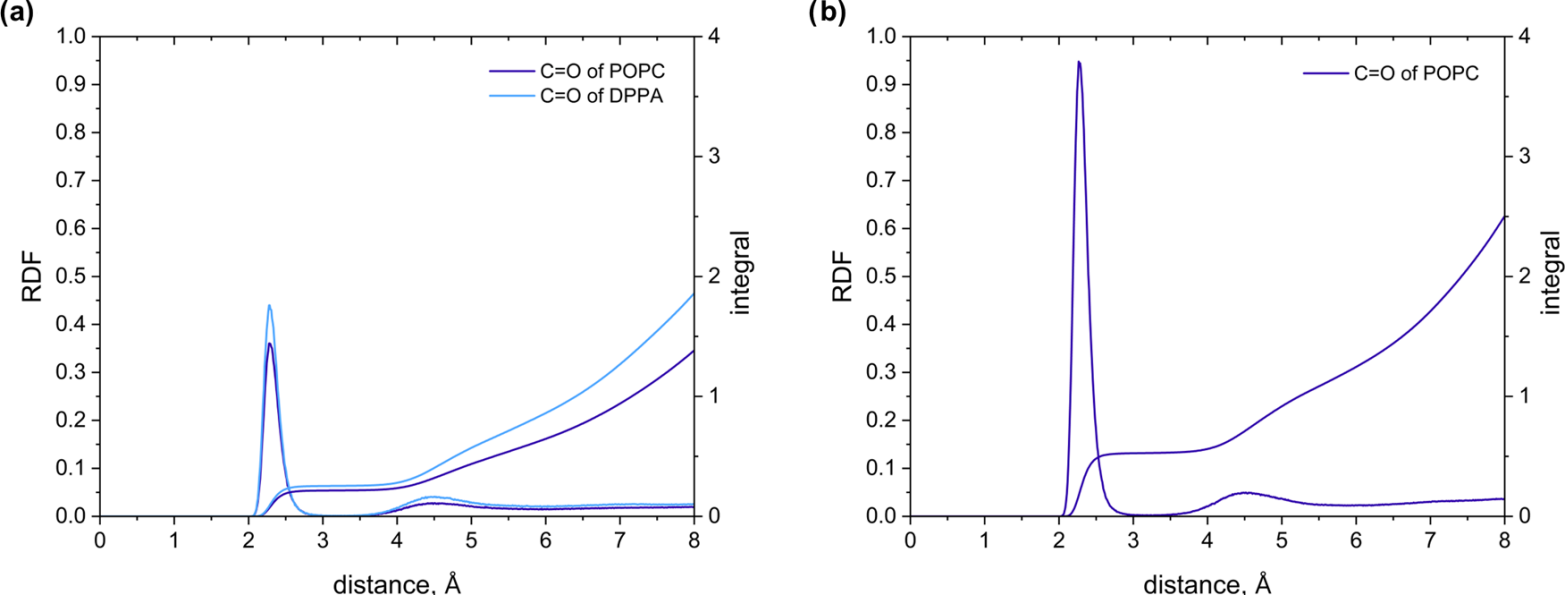
Radial distribution functions of Na^+^ ions around the carbonyl oxygen atoms of (a) the DPPA/POPC and (b) the DHDP/POPC systems.

To explain this difference, we additionally determined the RDFs for the pairs sodium cation–oxygen in the carbonyl moiety of the ester group (Figure 7). In the case of the DPPA/POPC system, the values of the RDF functions for POPC and DPPA are similar. In the DHDP/POPC system, the integral of the RDF for POPC is higher than the integral of the RDF for this lipid in the DPPA/POPC system, which is understandable. However, this increase is lower than the value of the integral of the RDF for DPPA in the DPPA/POPC system. This means that some amounts of sodium ions, which in the DPPA/POPC system are coordinated with the oxygen atoms in the carbonyl moiety of the ester group, must be coordinated in the DHDP/POPC system with the nonbridging oxygen atoms of the phosphate group. This coordination neutralizes the charge of the phosphate group in the POPC molecule, which affects the charge of the polar part of this phosphatidylcholine. Consequently, the electrostatic interactions between lipids are altered, leading to the repulsive interaction observed in Langmuir monolayer experiments conducted for the DHDP/POPC mixture. These results show that the presence of an ester group in the DPPA molecule makes it possible to maintain the electrostatic balance in systems with this lipid. Furthermore, this explains why the interactions in the DPPA/POPC system resemble those in the DPPC/POPC mixture.

We also confirmed the interchangeability of DPPC and DPPA by examining the conformational similarity of these molecules. For three systems of DPPC/POPC, DPPA/POPC, and DHDP/POPC mixtures, we determined the tilt angle of the P–N vector of the phosphocholine group relative to the normal interfacial plane. The average tilt angle of the P–N vector for POPC in the DPPC/POPC and DPPA/POPC systems is equal to 69.58 and 68.45°, respectively. A higher value of the tilt angle in the DHDP/POPC system, i.e., 80.17°, is caused by the small size of the DHDP polar group, which allows phosphatidylcholine to move more freely and orient almost parallel to the interfacial plane. This also translates to the values of the area per molecule. In the DPPC/POPC and DPPA/POPC systems, the area per molecule obtained from MD simulations is 61.89 and 57.53 Å^2^, respectively. In the DHDP/POPC system, the area per molecule is 45.64, which is the result of the overlap of phosphatidylcholine on the DHDP molecules.

Moreover, we compared the conformations of DPPC, DPPA, and DHDP by determining the dihedral angles for the polar groups. For the discussion, we used the notation from ref (60) (Figure S7 in the Supporting Information). The distribution of dihedral angles reveals the same conformation of the polar headgroup of POPC in the three systems, i.e., DPPC/POPC, DPPA/POPC, and DHDP/POPC (Figure S8 in the Supporting Information). α_1_ angle, which is the dihedral angle around the C_3_–O_31_ bond, is predominantly sin-clinical, which differs from the results obtained for POPC for the CHARMM36 and Berger force fields.^61^ The distributions of α_2_, α_3_, and α_5_ angles show similarity to those determined in simulations using the Berger and GAFFlipid force fields,^61,62^ while the α_4_ angle occurs ± sin-clinical ranges, which is ± (60 ± 30°), but also anti-periplanar (180 ± 30°), what appears to be the superposition of distributions determined previously for the CHARMM36 and Berger force fields.

The distribution of dihedral angles in the polar headgroup of DPPC in the DPPC/POPC system shows similarity to that of POPC (Figure S9 in the Supporting Information). The same applies to the α_1_ angle for DPPA, which is the dihedral angle around the C_3_–O_31_ bond. The distribution of the α_2_ angle for DPPA, an angle around the bridging O–P bond, also shows similarities to DPPC, however, with a slightly larger contribution of +sin-clinical instead of −sin-clinical angle. These distributions show the conformational similarity of DPPA to DPPC. In the case of DHDP, due to the lack of an ester group, the plot of α_1_ shows the equal distribution of the +sin-clinical and the -sin-clinical angles and the shift to the lower angle values (approximately 15°). The angles around the bridging O–P bond in DHDP are distributed, in turn, differently than in the case of DPPC and DPPA. The doublet distributions of the adjacent dihedral angles in the polar group of POPC and DPPC reveal sequences of sin-clinical/sin-clinical without a sign reverse (Figures S10 and S11 in the Supporting Information). This observation aligns with energy considerations.^63^ The exception is the pair α_1_/α_2_, where the sequence ±sin-clinical/∓sin-clinical is more significant and in the case of the DPPC/POPC and DHDP/POPC predominates.

A comparison of the dihedral angles for the glycerol backbone also shows a similar conformational behavior of POPC in all of the systems analyzed (Figure S12 in the Supporting Information). In addition, in the case of DPPC and DPPA, these angles point to conformational similarity to POPC (Figure S13 in the Supporting Information). The mutual orientation of the *sn-1* and *sn-2* chains is specified by the θ_4_ dihedral angle (the dihedral angle around the C_1_–C_2_ bond), while the θ_2_, around C_2_–C_3_, determines how the headgroup aligns in relation to the *sn-2* chain. For all lipid glycerol backbones, the distributions of θ_4_ (and its complement for tetrahedral bond angles θ_3_–120°) show the presence of only ± sin–clinical angles, which is the condition of parallel chain stacking characteristic for lipid membrane arrangement.^61^ The doublet distribution of the pairs θ_2_/θ_4_ (Figure S14 in the Supporting Information) shows the predominant presence of +sin–clinical/anti-periplanar configuration with a diminishing (except for DPPA) contribution from +sin–clinical/+sin–clinical relation and negligible presence of +sin–clinical/-sin–clinical combination. The latter is disfavored because that would rotate the headgroup back into the layer.

In summary, the magnitude of interactions between PCs (in this case, DPPC and POPC) results mainly from the contribution of polar interactions from phosphate and glycerol groups in phospholipid polar heads. This is evidenced by molecular dynamics simulations, which have shown that the absence of the ester group in the molecule implies a change in the coordination of the phosphate group of the phospholipid, disrupting interlipid interactions. The similarities between DPPC/POPC and DPPA/POPC systems in terms of subphase anchoring, coordination shell, electrostatic balance, and polar head tilt angles also confirm this deduction. This leads to the conclusion that the presence of both ester and phosphate groups is crucial for maintaining the electrostatic balance in monolayer lipid systems.

## 4 Conclusions

The study was aimed at finding structural motives in PC molecules (particularly individual polar groups) that are of fundamental importance in maintaining the biophysical properties of the cellular membrane. To achieve this, we have selected two-component mixtures of PCs (DPPC/POPC) and PC/Chol because these compounds are seen as the main membrane lipids. In a systematic way, we examined the interactions and rheological properties of a number of systems containing commercially available synthetic PC analogues, in which one or two polar groups present in PC were eliminated. This allowed us to identify the ternary amine and phosphate groups as those responsible for interactions in PC/cholesterol and PC/PC systems, respectively. Moreover, the importance of the esterified glycerol moiety as a modulator of the interaction magnitude was shown. These findings contribute to basic knowledge as they establish structure–property relationships for the main membrane lipids. Additionally, the knowledge achieved may find application in the design of laboratory experiments involving synthetic analogues instead of natural phosphatidylcholines, which can be more expensive and less stable. However, it should be emphasized that PC analogues are not universal and that the choice of a specific compound is dictated by the content of other membrane components. The developed methodology can be useful not only to mixtures with natural or synthetic PCs but also to molecules of biomedical importance having a phospholipid-like structure, like, for example, new generation antitumor lipids (ATLs).

## Supporting Information Available

Surface pressure-molecular area isotherms; compressibility moduli surface pressure curves; excess Gibbs free energy of mixing mole fraction curves; and notation and distribution of dihedral angles (PDF)
