# Supplementary material for: Insight into the Molecular Mechanism of Surface Interactions of Phosphatidylcholines—Langmuir Monolayer Study Complemented with Molecular Dynamics Simulations
Source: J Phys Chem B. 2024 Feb 6;128(6):1473–82. doi: 10.1021/acs.jpcb.3c06810 (PMC10875670; doi:10.1021/acs.jpcb.3c06810)
Supplement: Supplementary file 1 — jp3c06810_si_001.pdf [file jp3c06810_si_001.pdf]

Supporting Information

for

Insight into the Molecular Mechanism of Surface Interactions of Phosphatidylcholines - Langmuir Monolayer Study Complemented with Molecular Dynamics Simulations

Anna Chachaj–Brekiesz[1,*], Jan Kobierski[2], Anita Wnętrzak[1],
Patrycja Dynarowicz–Latka[1], Patrycja Pietruszewska[1]

[1] Faculty of Chemistry, Jagiellonian University, Gronostajowa 2, 30–387 Kraków, Poland
[2] Department of Pharmaceutical Biophysics, Faculty of Pharmacy, Jagiellonian University Medical College, Medyczna 9, 30–688 Kraków, Poland

* corresponding author, e–mail: anna.chachaj@uj.edu.pl

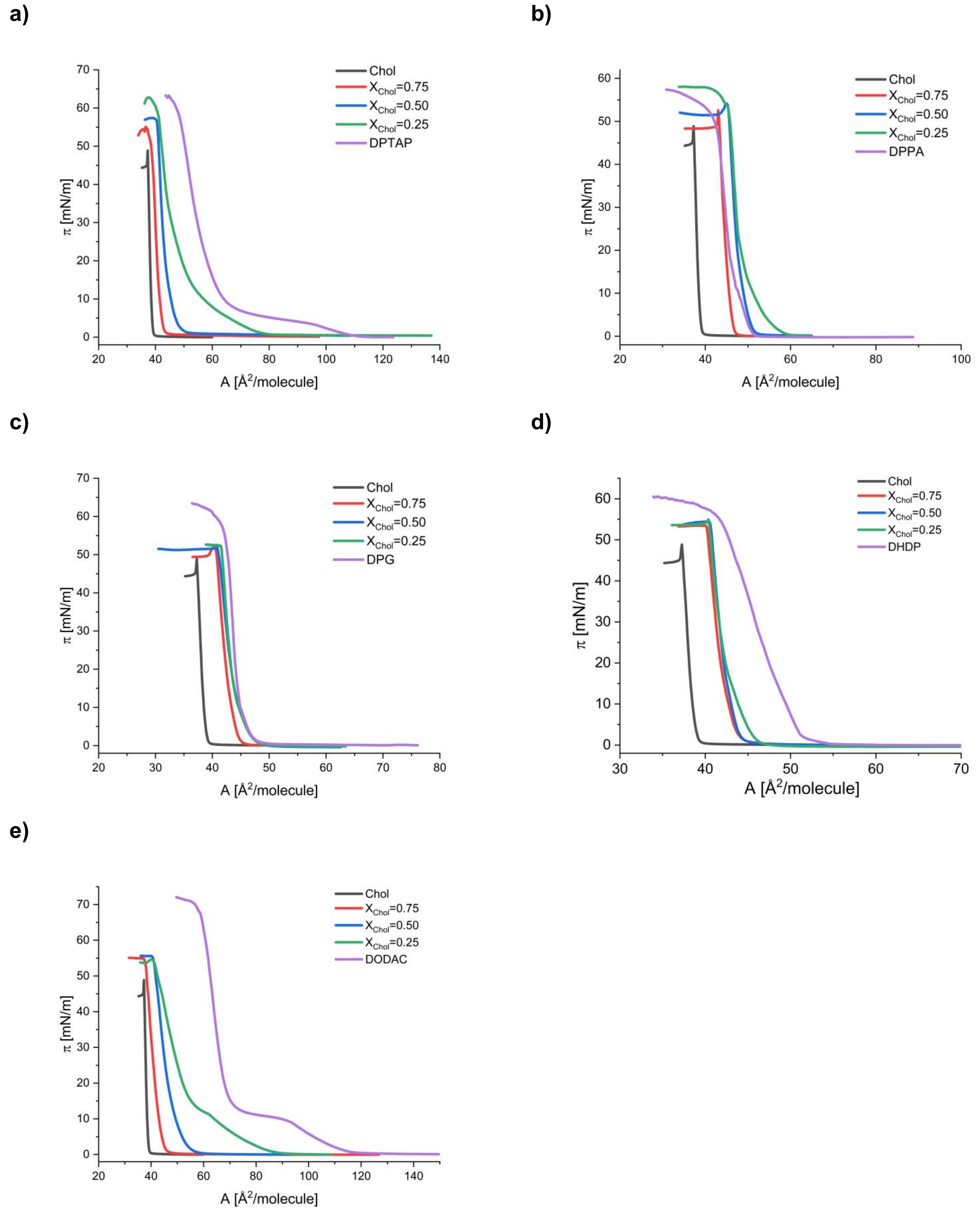

Figure S1. Experimental surface pressure – area per molecule isotherms measured at 20°C for mixed systems of cholesterol and (a) DPTAP, (b) DPPA, (c) DPG, (d) DHDP, (e) DODAC.

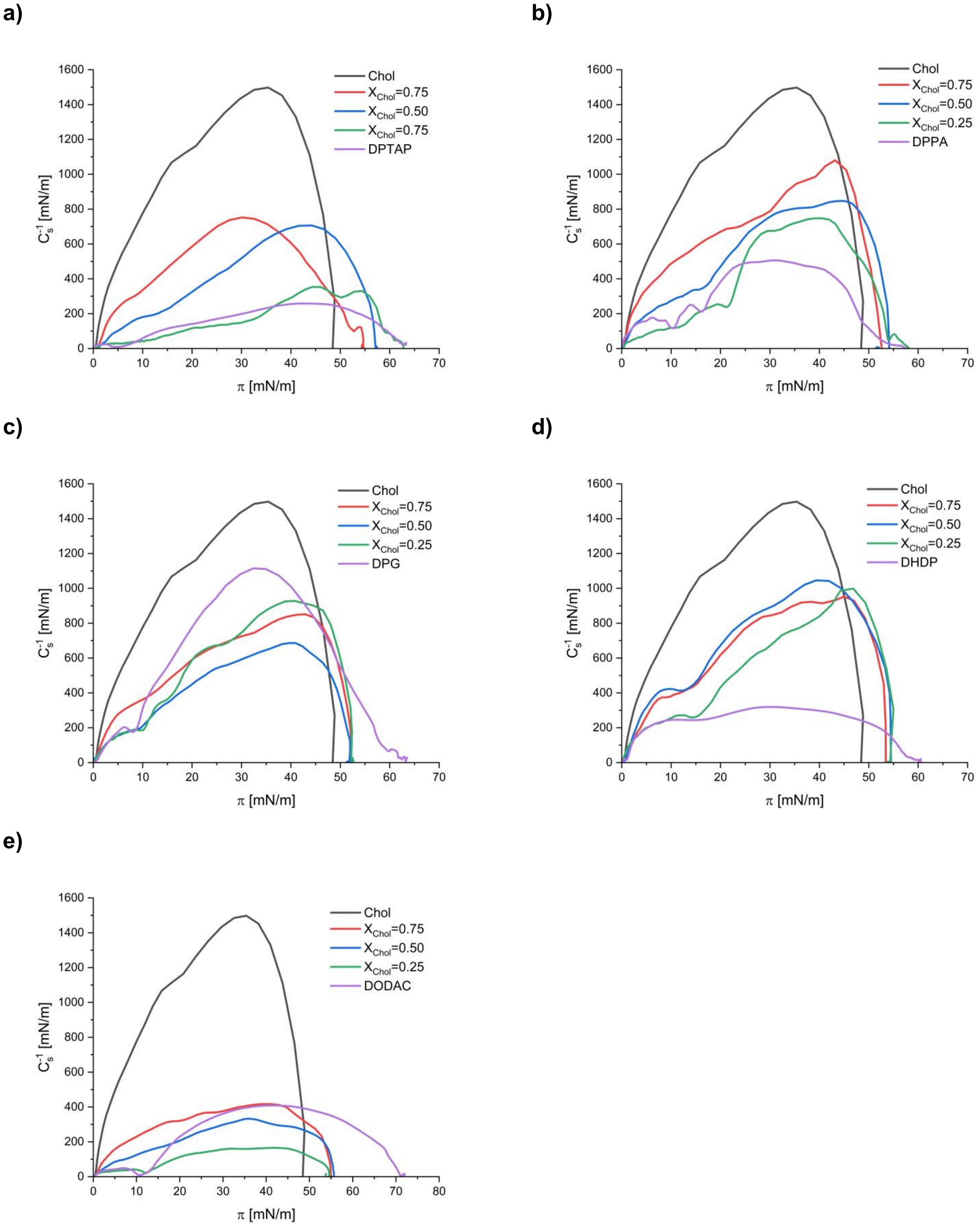

Figure S2. Calculated compressibility moduli – surface pressure curves for mixed systems of cholesterol and (a) DPTAP, (b) DPPA, (c) DPG, (d) DHDP, (e) DODAC.

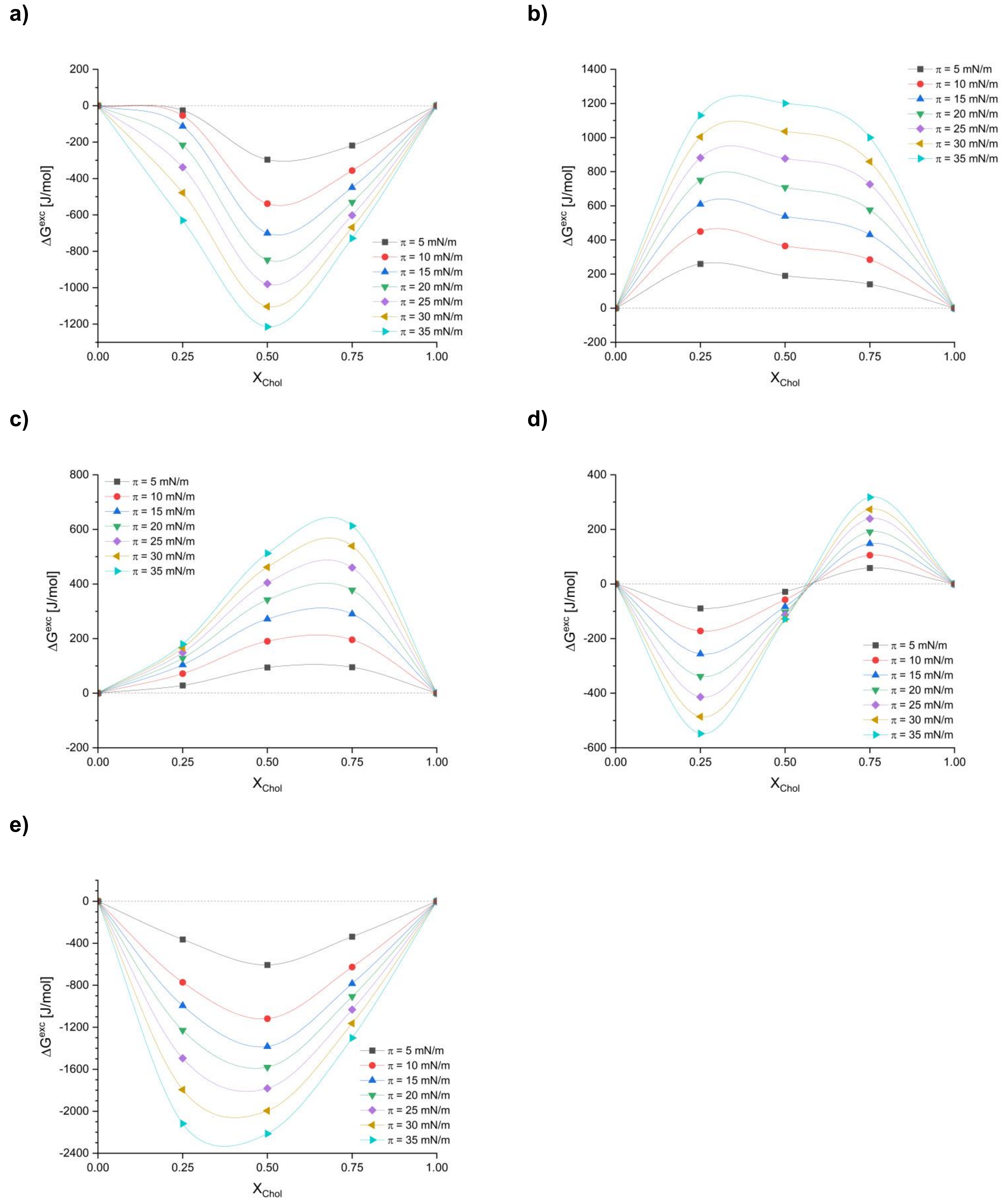

Figure S3. Calculated excess Gibbs free energy of mixing – cholesterol mole fraction curves at selected surface pressure values for mixed systems of cholesterol and (a) DPTAP, (b) DPPA, (c) DPG, (d) DHDP, (e) DODAC.

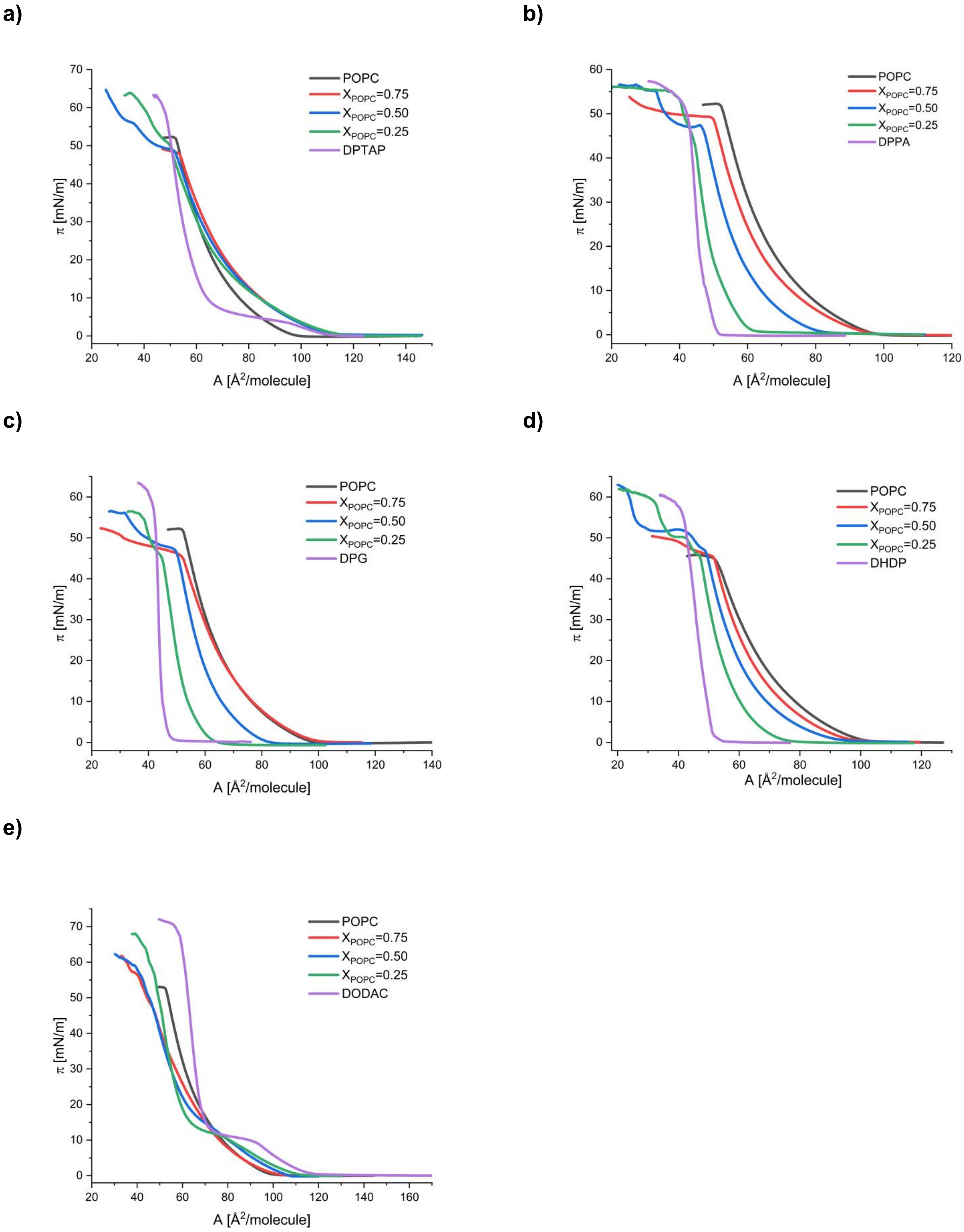

Figure S4. Experimental surface pressure – area per molecule isotherms measured at 20°C for mixed systems of POPC and (a) DPTAP, (b) DPPA, (c) DPG, (d) DHDP, (e) DODAC.

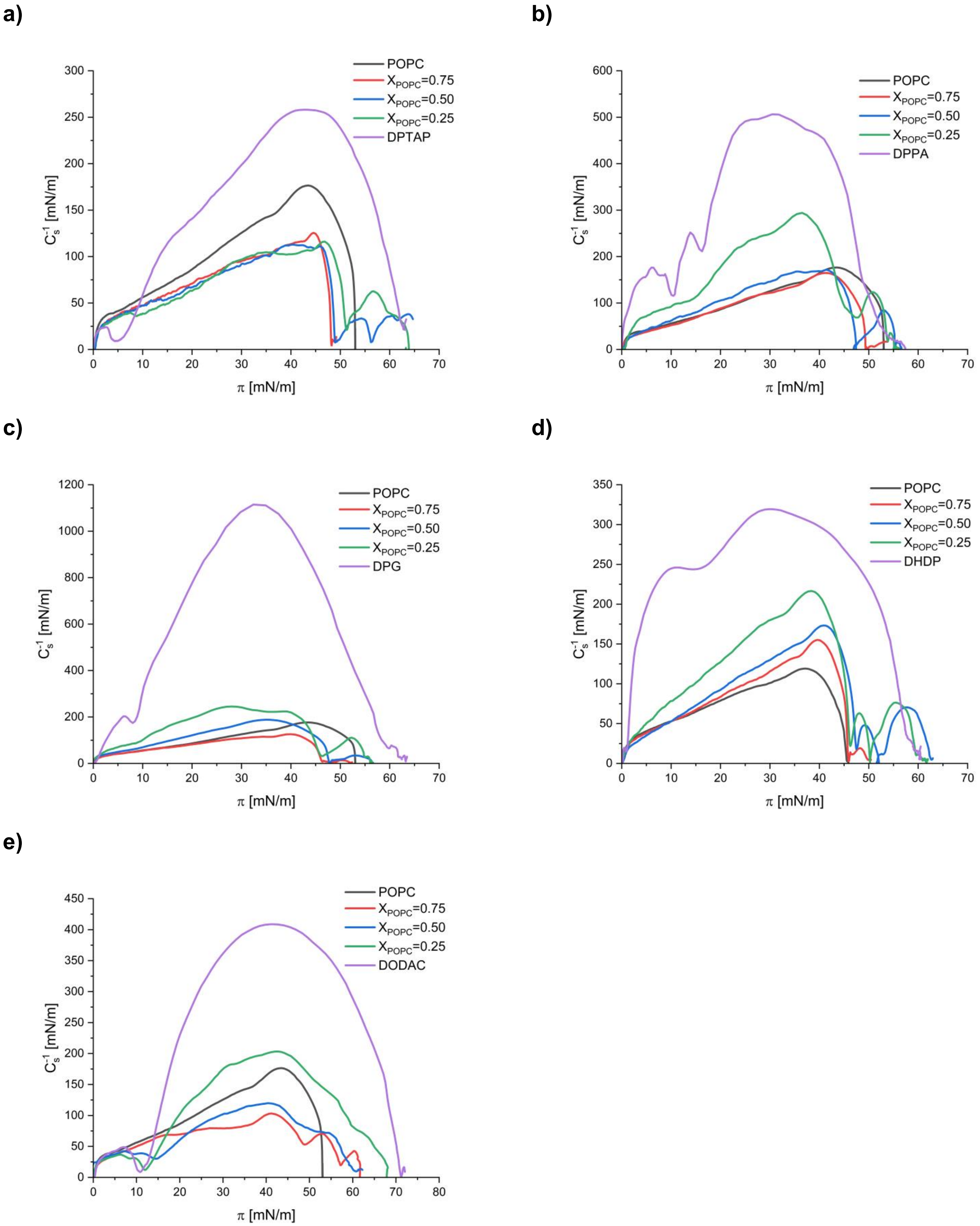

Figure S5. Calculated compressibility moduli – surface pressure curves for mixed systems of POPC and (a) DPTAP, (b) DPPA, (c) DPG, (d) DHDP, (e) DODAC.

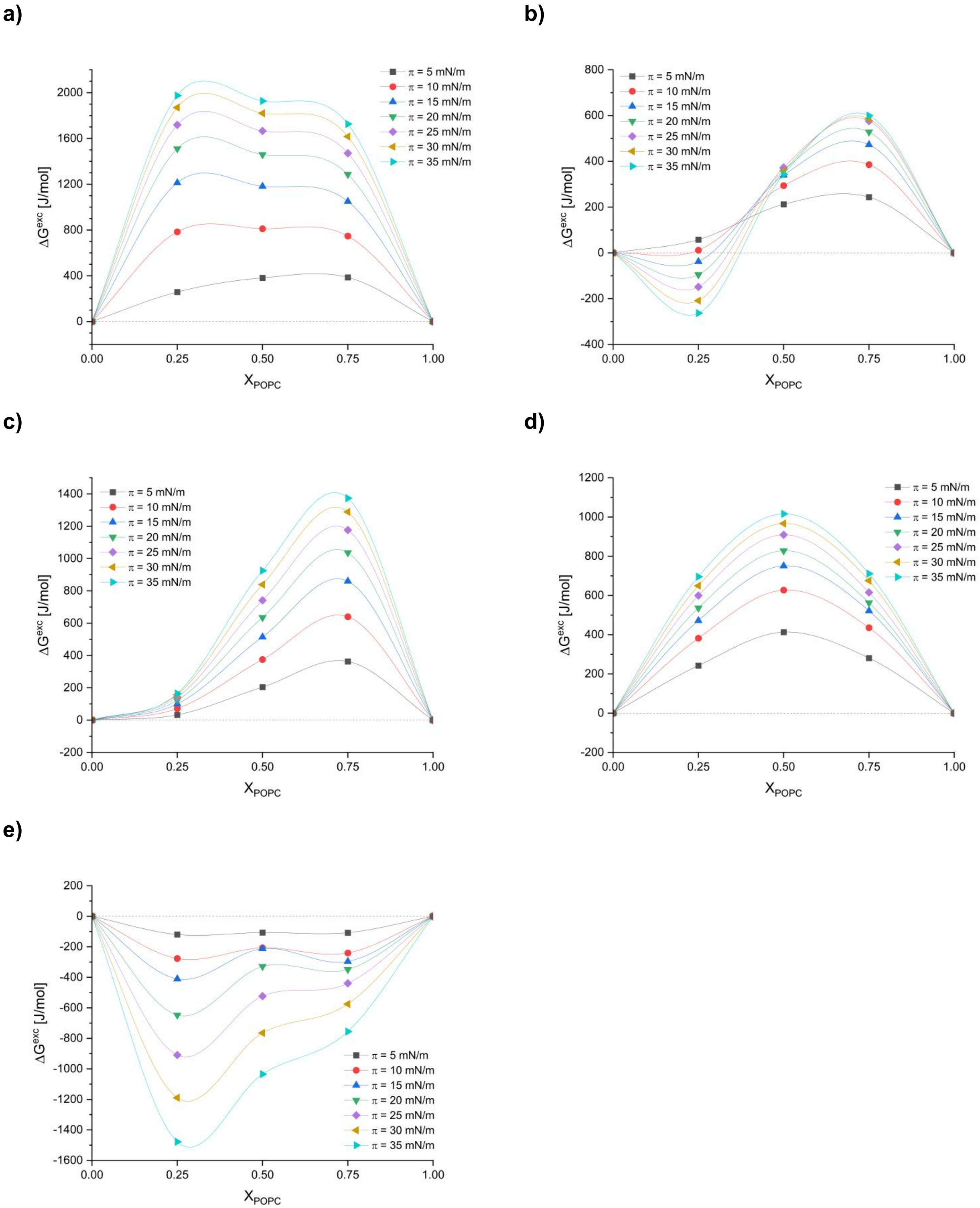

Figure S6. Calculated excess Gibbs free energy of mixing – POPC mole fraction curves at selected surface pressure values for mixed systems of POPC and (a) DPTAP, (b) DPPA, (c) DPG, (d) DHDP, (e) DODAC.

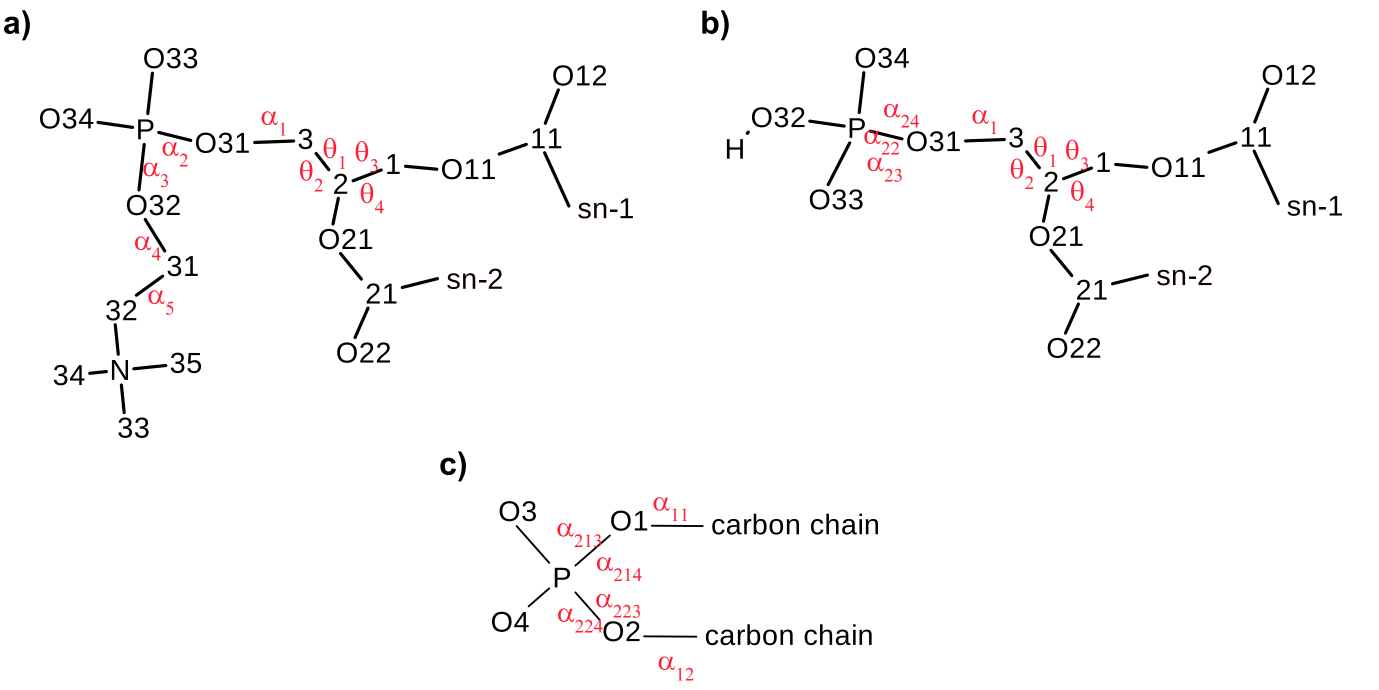

Figure S7. Notation of dihedral angles in (a) phosphatidylcholines, (b) DPPA, and (c) DHDP.

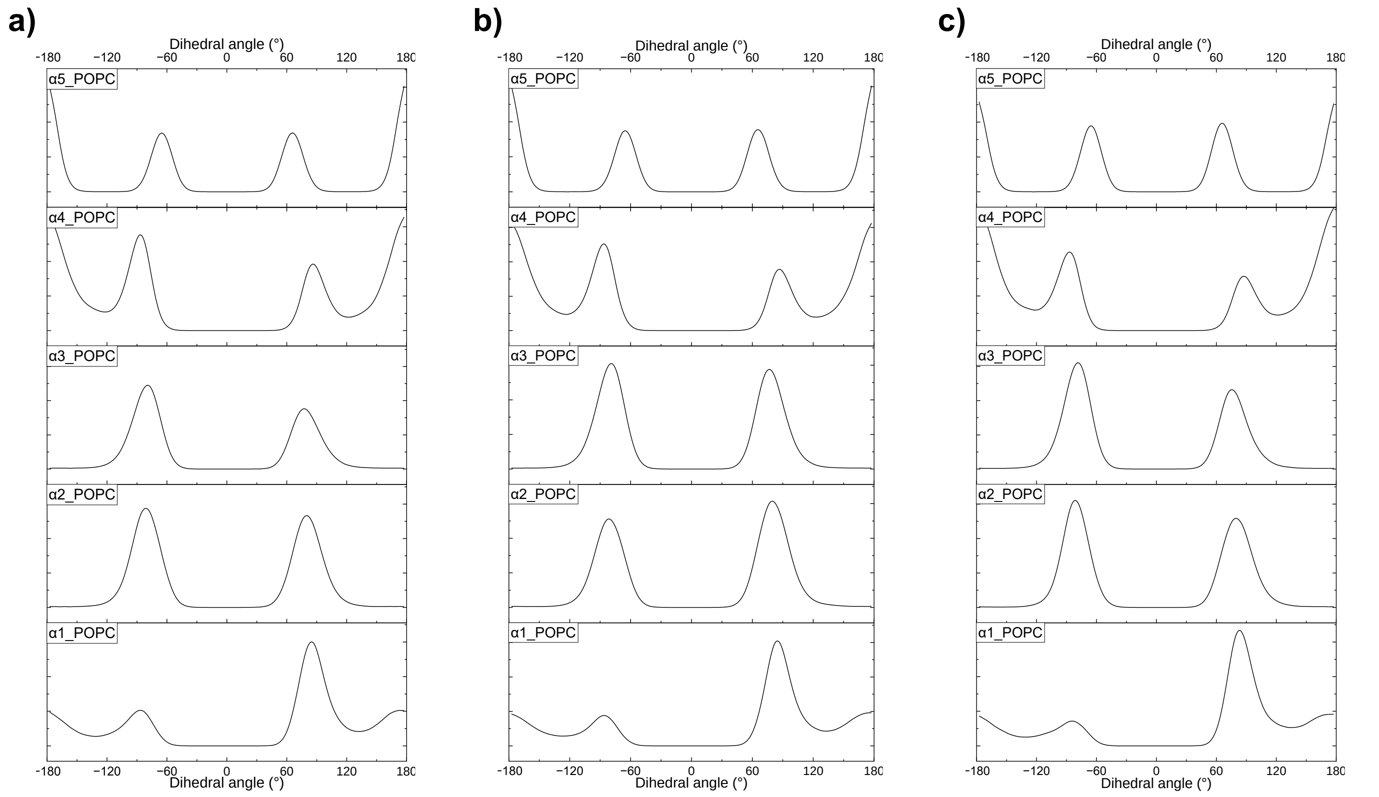

Figure S8. Distribution of dihedral angles in the polar headgroup of POPC in mixed systems: (a) DPPC/POPC, (b) DPPA/POPC, and (c) DHDP/POPC.

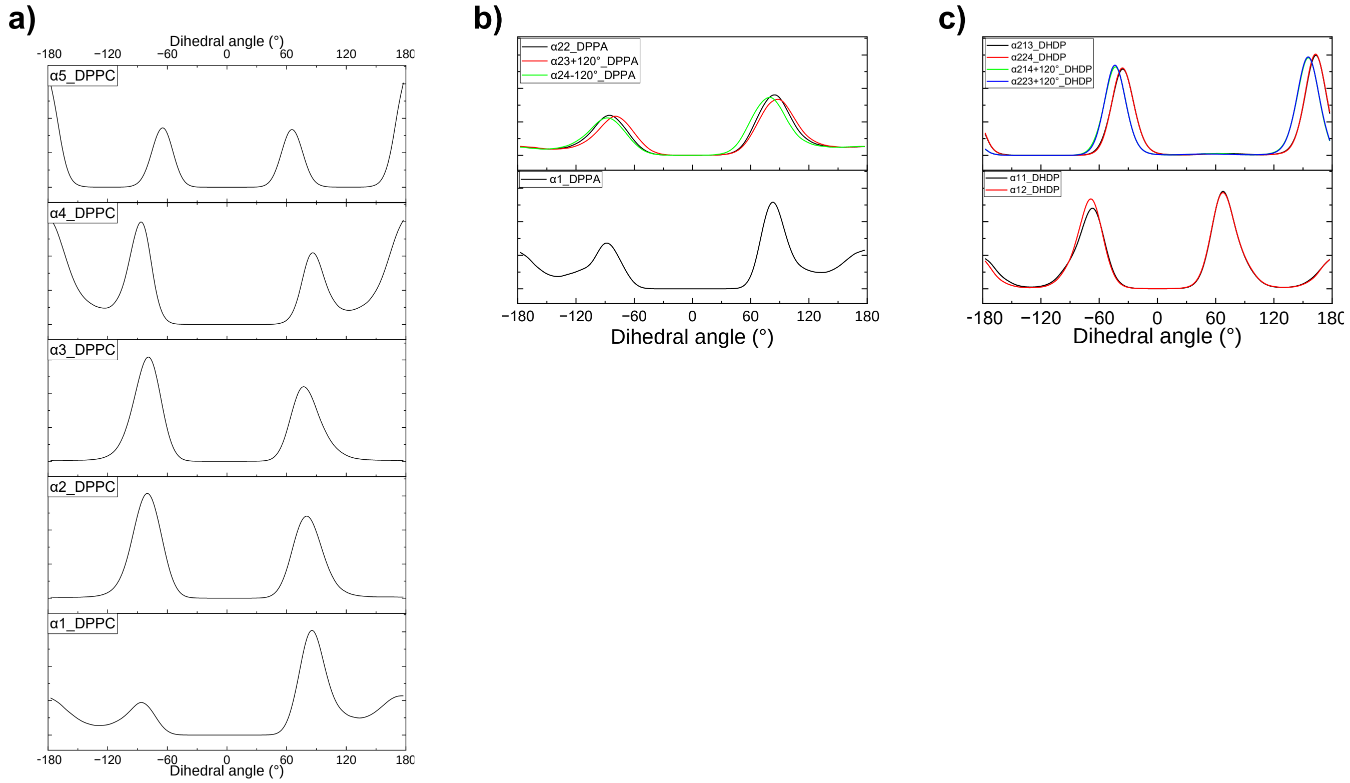

Figure S9. Distribution of dihedral angles in the polar headgroup of (a) DPPC, (b) DPPA, and (c) DHDP in mixed systems with POPC.

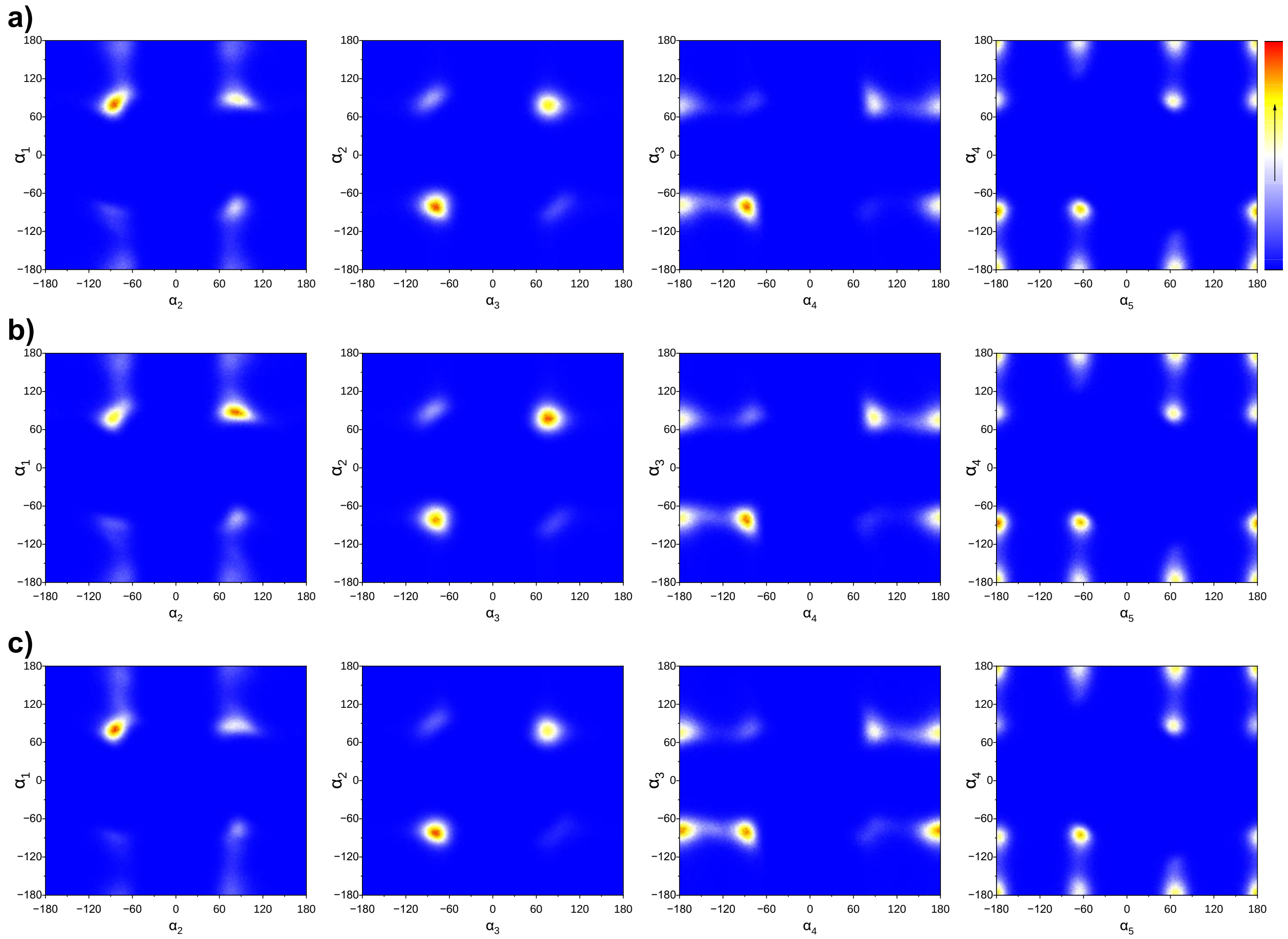

Figure S10. Doublet distribution of dihedral angles pairs in the headgroup of POPC in the mixed systems: (a) DPPC/POPC, (b) DPPA/POPC, and (c) DHDP/POPC.

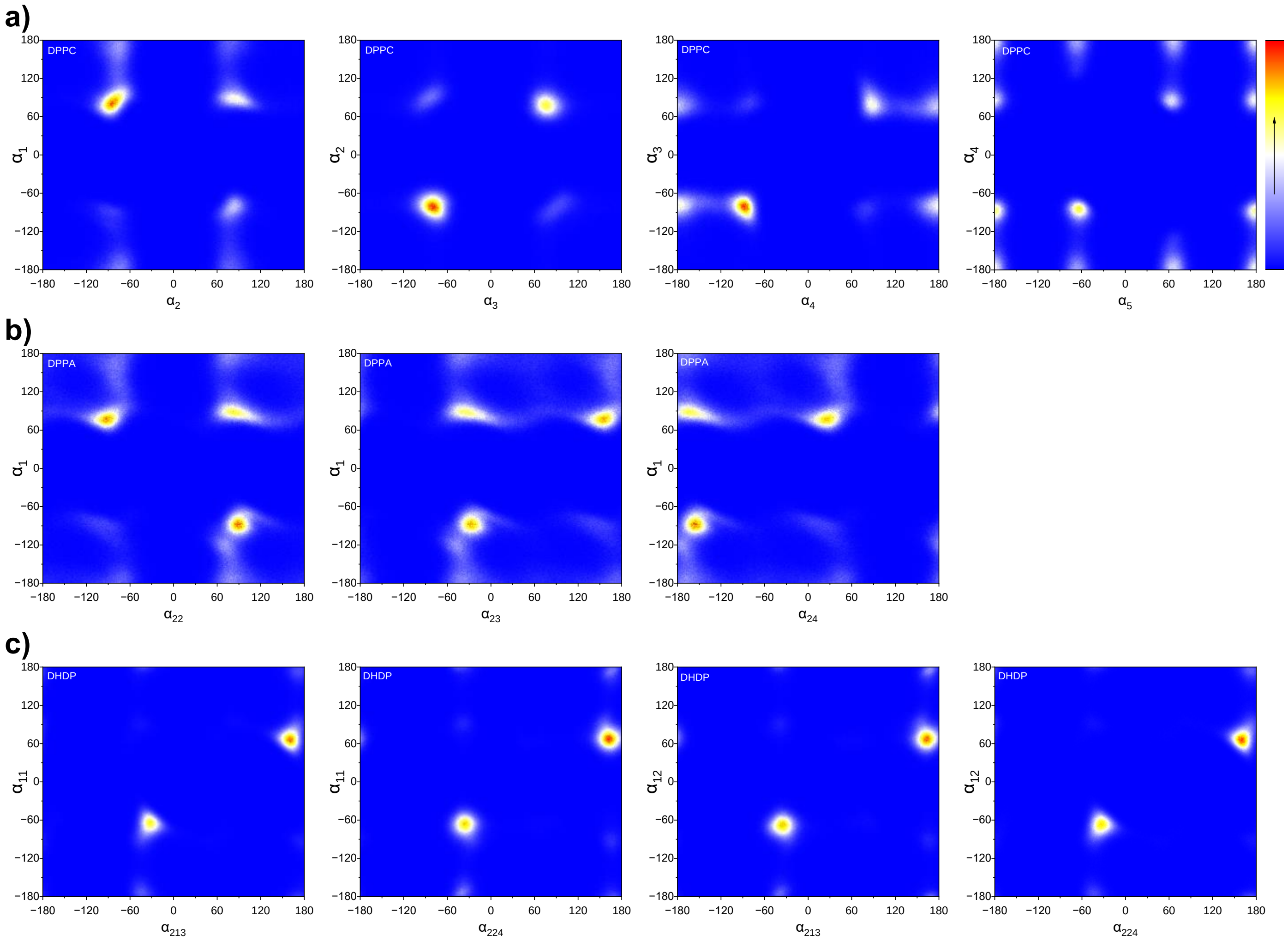

Figure S11. Doublet distribution of dihedral angles pairs in the headgroup of (a) DPPC, (b) DPPA, and (c) DHDP in the systems with POPC.

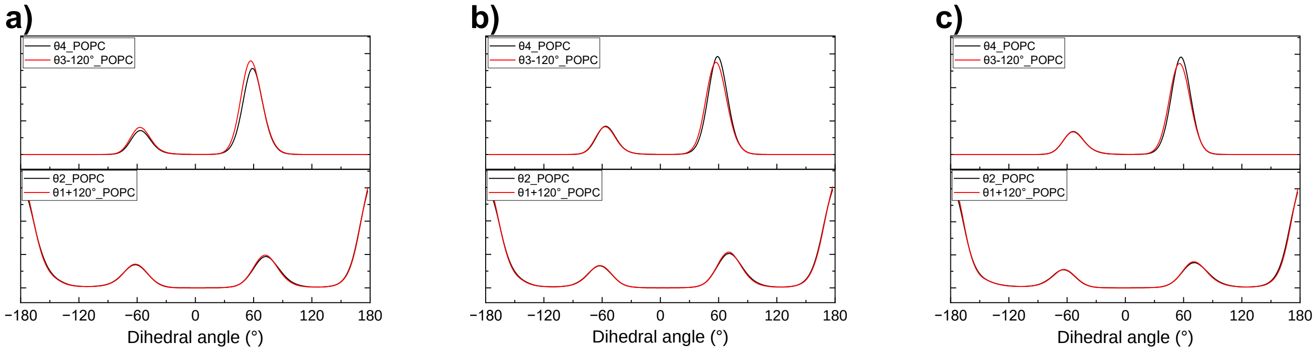

Figure S12. Distribution of dihedral angles about the C2-C3 bond (θ_4 and θ_3-120°) and C1-C2 bond (θ_2 and θ_1-120°) in the glycerol backbone of POPC in mixed systems: (a) DPPC/POPC, (b) DPPA/POPC, and (c) DHDP/POPC.

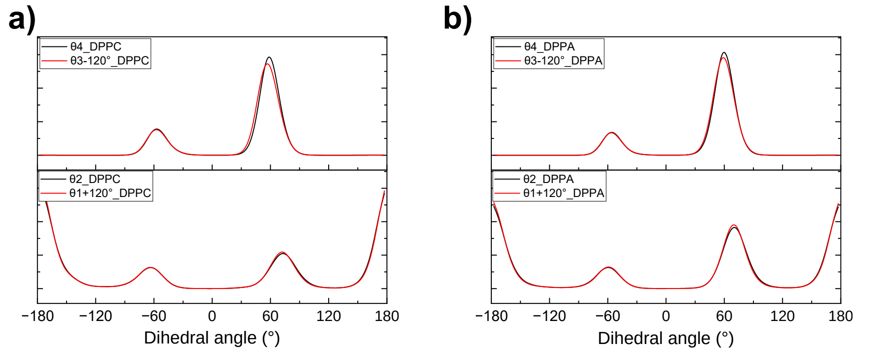

Figure S13. Distribution of dihedral angles about the C2-C3 bond (θ_4 and θ_3-120°) and C1-C2 bond (θ_2 and θ_1-120°) in the glycerol backbone of (a) DPPC and (b) DPPA in mixed systems with POPC.

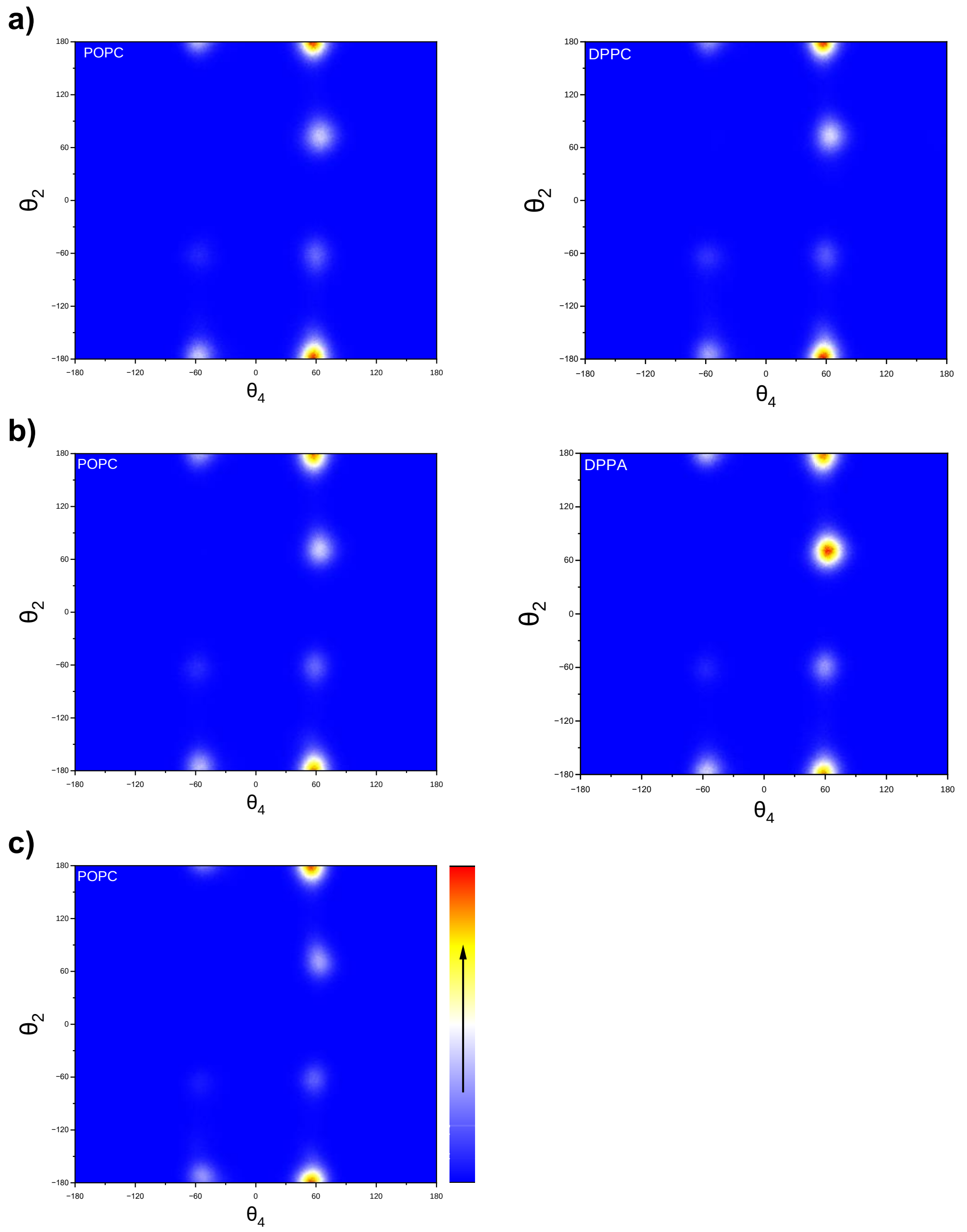

Figure S14. Doublet distribution of dihedral angles pairs θ_2/θ_4 in the glycerol backbones for the systems: (a) DPPC/POPC, (b) DPPA/POPC, and (c) DHDP/POPC.
